# Re-examining balinese subaks through the lens of cultural multilevel selection

**DOI:** 10.1007/s11625-017-0453-1

**Published:** 2017-08-07

**Authors:** Jeremy Brooks, Victoria Reyes-García, William Burnside

**Affiliations:** 1 School of Environment and Natural Resources, The Ohio State University, Columbus, OH USA; 2 ICREA, Passeig de Lluis Companys 23, 08010 Barcelona, Spain; 3 Institut de Ciència i Tecnologia Ambientals, Universitat Autònoma de Barcelona, Bellatera, 08193 Barcelona, Spain; 4 National Socio-Environmental Synthesis Center, Annapolis, MD 21403 USA

**Keywords:** Social–ecological systems, Cultural multilevel selection, Common pool resource, Cultural transmission, Collective action, Sustainability

## Abstract

Overcoming environmental challenges requires understanding when and why individuals adopt cooperative behaviors, how individual behaviors and interactions among resource users change over time, and how group structure and group dynamics impact behaviors, institutions, and resource conditions. Cultural multilevel selection (CMLS) is a theoretical framework derived from theories of cultural evolution and cultural group selection that emphasizes pressures affecting different levels of social organization as well as conflicts among these levels. As such, CMLS can be useful for understanding many environmental challenges. With this paper, we use evidence from the literature and hypothetical scenarios to show how the framework can be used to understand the emergence and persistence of sustainable social–ecological systems. We apply the framework to the Balinese system of rice production and focus on two important cultural traits (synchronized cropping and the institutions and rituals associated with water management). We use data from the literature that discusses bottom-up (self-organized, complex adaptive system) and top-down explanations for the system and discuss how (1) the emergence of group structure, (2) group-level variation in cropping strategies, institutions, and rituals, and (3) variation in overall yields as a result of different strategies and institutions, could have allowed for the spread of group-beneficial traits and the increasing complexity of the system. We also outline cultural transmission mechanisms that can explain the spread of group-beneficial traits in Bali and describe the kinds of data that would be required to validate the framework in forward-looking studies.

## Introduction

Environmental challenges, including the sustainable management of common pool resources (CPR), share three features. First, they are often the result of social dilemmas, or situations in which the interests of the individual conflict with those of the group (Gardner and Stern 1996). Second, they are dynamic; individuals and institutions must adapt to social and environmental conditions creating feedback loops between social and ecological systems. Third, environmental challenges are often embedded in social–ecological systems containing multiple groups and exhibiting hierarchical group structure (e.g., households, villages, communities, state/regional governments, etc.) (Ostrom 2010). Given these features, overcoming environmental challenges requires understanding when and why individuals adopt cooperative behaviors to overcome social dilemmas, how individual behaviors and interactions among resource users change over time, and how group structure and dynamics (including competition within and between levels in a nested hierarchy) impact the coevolution of behaviors, institutions, and resource conditions.

There has been extensive research on the conditions that enable sustainable CPR management (Agrawal 2003; Ostrom 2009), and numerous conceptual and analytical frameworks have been used to structure our understanding of human-environmental systems and their governance [e.g., resilience (Folke 2006), social–ecological systems (Ostrom 2009), coupled human and natural systems (Liu 2007), multilevel governance frameworks (Pahl-Wostl 2009)]. However, generalizable insights into the causal mechanisms affecting change across organizational levels have been elusive (Waring et al. 2015). Such insights are critical for improving our understanding of when and how sustainable social–ecological systems emerge and persist. Here, we aim to demonstrate that a cultural multilevel selection framework can provide important insights about the evolution of social–ecological systems.

Cultural multilevel selection (CMLS) is a theoretical framework that focuses on cultural evolutionary processes (e.g., variation, selection, transmission) occurring when human populations are structured in social groups (Boyd and Richerson 1985; Wilson and Wilson 2007). CMLS emphasizes not only the pressures affecting groups at different levels of social organization, but also the importance of understanding conflict among these levels. For example, individuals are embedded within kin groups, which are embedded within societies, but the interests of individuals, kin groups, and societies are not always aligned (Reyes-García et al. 2016). CMLS might be particularly useful for understanding environmental challenges that require repeated cooperative acts of many individuals, sometimes from socially distinct and geographically distant groups. By applying evolutionary concepts that focus on adaptive change to group structure, CMLS can help us better understand how, when, and where solutions to environmental social dilemmas emerge and proliferate (Waring et al. 2015).

However, the application of the CMLS framework to environmental challenges is its infancy. As such, our primary objectives are: (1) to demonstrate how to apply the CMLS framework to understand the dynamics of a sustainable social–ecological system, (2) to outline the causal mechanisms that could have contributed to the emergence and spread of collective action in this system, and (3) to highlight the data that would be required to fully test hypotheses derived from the CMLS framework about the evolution of social–ecological systems. To meet these objectives, we re-examine the classic agro-ecological system in Bali.

Bali is well-known for a characteristic, ritualized system of rice production (Geertz 1980; Lansing 2006; Wittfogel 1957) that allows farmers to navigate environmental tradeoffs and constraints (Hakim et al. 2009). The construction of irrigation canals and the existence of nested institutions for coordinating irrigation and cropping schedules across the landscape has created a culturally rich and ecologically productive system that is at least 1000-years-old (Lansing 2006). Importantly, the system requires few inorganic inputs. Fields benefit from natural fertilization as rainwater extracts minerals from volcanic rock and the coordination of irrigation patterns prevents the spread of pests, reducing the need for pesticides. Intriguingly, the nested institutions that coordinate irrigation and cropping patterns align interests at different hierarchical levels, a situation that has resulted in relatively equitable yields for farmers in the same group (Lansing 2006; Lansing and Fox 2011). As a long-lived system with clear group structure, rice production in Bali is an ideal case for illustrating how the CMLS framework can inform our understanding of the emergence and persistence of social–ecological systems.

Extensive research has documented key components of the system and produced critical insights into how it functions (e.g., Lansing 1987, 2006; Schoenfedler 2001; Scarborough et al. 1999). In addition, agent-based models have been used to suggest a general mechanism through which the system could have self-organized (Lansing and Kremer 1993). However, gaining insight into how particular behaviors, organizational structures, and institutions emerged and spread across the Balinese landscape has been difficult in part due to the system’s longevity and gaps in the written and archeological record. Given these data limitations, we are compelled to rely on hypothetical scenarios to describe mechanisms operating at multiple scales that could have contributed to the emergence and spread of the system. We use two scenarios, supported where possible by direct evidence, to mirror the two primary scholarly arguments about the origins and management of the system, one suggesting that Bali’s agro-ecological system emerged from the bottom-up through a process of self-organization (Lansing 2006) and the other suggesting that it was historically shaped and controlled from the top-down by royal elites (Hauser-Schäublin 2003; Schulte Nordholt 2011). We conclude by outlining the data that would allow us to test more fully the validity of the CMLS framework for explaining this case and for understanding the structure and dynamics of contemporary social–ecological systems.

## Cultural multilevel selection framework

The CMLS framework derives from theories of cultural evolution (Boyd and Richerson 1985) and cultural group selection (Henrich 2004). Cultural evolution is based on evolutionary logic applied to cultural traits, which we define as socially transmitted behaviors, norms, beliefs, attitudes, and institutions. This theory posits that individuals learn strategically from others, preferentially copying behaviors or attitudes from those whose traits appear to confer an advantage (Mesoudi 2011). Differential imitation acts as a form of natural selection, changing the prevalence of cultural traits in a population over time. Through this process, human behaviors and institutions co-evolve with the local environment.

Individual imitation, however, cannot always explain the spread of behaviors that are costly to the individual but beneficial to the group, such as cooperation or other behaviors that are needed to solve social dilemmas. Cultural group selection is a general theory that can explain the spread of cooperative behaviors (Henrich 2004; Richerson et al. 2016). When clearly defined groups vary in a trait that affects their success, selection can act to favor groups exhibiting a group-beneficial trait even if this trait is costly to individuals (Boyd and Richerson 2009). For instance, a large company whose employees work unselfishly might earn more business than another company whose selfish employees compete with each other, even though employees in the first company sacrifice individual success in the process. Through this mechanism, individually costly but group-beneficial behaviors can persist and spread (Richerson et al. 2016).

The CMLS framework suggests that group-beneficial behaviors will be favored when selection pressures between groups are stronger than selection pressures among individuals. Group selection is most likely to act on a group-beneficial trait (i.e., cooperation), thus resulting in the spread of the trait among groups, when: (1) the trait produces group-level benefits that exceed individual costs, (2) there is greater variation in expression of the trait among groups than within groups, and (3) the proliferation (or decline) of the trait is directly related to the outcomes it produces (Price 1972; Rogers 1990).

Derived from these principles, the application of the CMLS framework to an environmental dilemma should specify: the individual-level costs and group-level benefits associated with a potentially relevant group-beneficial cultural trait and the group structure and group-level variation in trait expression (Waring et al. 2015). Moreover, the history and dynamics of selection pressures can help us grasp trait emergence and spread. We outline these components of Bali’s agro-ecological system below.

## Balinese agro-ecology: the subak as a response to social dilemmas

**Fig. 1:**
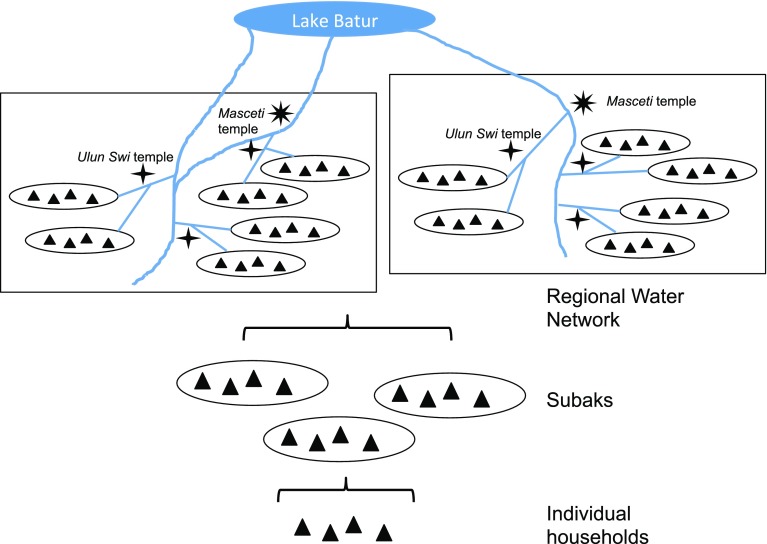
Organizational structure. Simplified diagram of the organizational structure for coordinating wet-rice cultivation in Bali from the bottom-up perspective. Individual farmers plant and harvest their own crops. Farmers are clustered in subaks, which coordinate cropping among households sharing the same water source. Subaks are clustered in regional water networks, which coordinate cropping patters among them

Decades of research have uncovered a set of highly ritualized institutions that have helped Balinese farmers solve the social dilemmas associated with managing water and coordinating cropping patterns for the sustainable production of rice. At the base of this set of institutions are subaks, or associations of farmers who own land irrigated by a common water source (Lansing 2006) (see Fig. 1). Subaks are nested within regional water networks, which contain water temples located at periodic nodes near the rivers and irrigation channels that weave across the island. Local water temples are subordinated to regional water temples such that the cultural practices that facilitate coordination are reflected in a nested hierarchy of farmers groups and water temples (Lansing 2006).

The maintenance of this sustainable system in Bali requires collective action. Decisions about water use and cropping patterns constitute a social dilemma. Each farmer has an incentive to withdraw as much water as possible to maximize yields (Scarborough et al. 1999), yet unrestricted water use by upstream farmers can lead to downstream water scarcity. However, if farmers irrigate at different times to reduce water stress, the resulting mosaic of wet and dry fields allows pests (insects and rodents) and plant diseases to thrive. Coordinating periods of flood and fallow over a sufficiently large area deprives pests of food and habitat, but it requires farmers to share water so planting can occur at the same time upstream and downstream. Thus, cooperation and coordination are needed to balance individual water needs for rice growth with the strategic use of water to prevent pests from spreading.

## The CMLS framework applied to subaks

Here, we describe the costs and benefits of two interrelated cultural traits that might generate group benefits. The first trait, synchronized cropping, is a cooperative trait expressed at the individual-level. The second trait, a complex of institutions, rituals, and rites associated with subaks and regional water temples, is expressed at the group-level (Smaldino 2014).

### Trait 1: synchronized cropping

The two main ecological constraints for rice production in Bali, water scarcity and pest damage, can be overcome by adopting either a staggered or a synchronized cropping pattern. When water is limited, it is in the best interest of upstream and downstream farmers to stagger their cropping (Lansing and Miller 2005) to spread peak water demand over a larger period of time. However, when pest damage is the greater threat to yields, it is in the best interest of both farmers to synchronize their cropping so that fields are flooded and fallowed at roughly the same time.

Both cropping strategies require collective action. Indeed, the individual costs and benefits of these strategies have been described as a two-player game-theoretic problem that is best solved when the upstream and downstream farmers coordinate their actions (Lansing and Miller 2005). Presumably because pest damage is a greater threat to yields than water stress in Bali (Scarborough et al. 1999), synchronization is the most common cropping pattern (Janssen 2007; Lansing and Kremer 1993). So, we focus on this variant of the trait for the remainder of the paper.

### Trait 2: institutions, rites, and rituals

The second cultural trait is the set of institutions, rites, and rituals that govern water use and influence collective action. Formal institutions and complex religious rituals are important in Bali for minimizing free-riding (water theft), facilitating coordination and collective action, and providing a cultural foundation for maintaining group solidarity.

Formal institutions, i.e., subaks, are important for reducing water theft and maintaining infrastructure. The existence of upstream and downstream farmers within a group as well as upstream and downstream groups of farmers creates the incentive for individual farmers to free-ride (Lansing and Miller 2005). An individual upstream farmer can take more than his fair share of water because such cheating will have little impact on the downstream group (or on pest populations) provided that the remaining upstream farmers share. Lansing (2006: 67) notes that “…farmers in the upper subaks admit that they are often tempted to take a little more water. But such cheating is rare and usually occurs only in the tiny canals…” To minimize cheating, subaks use fines and other penalties to sanction infractions such as stealing water and missing subak meetings or work assignments. The existence of more severe penalties like having one’s water supply cut off or being expelled from the subak altogether (Lansing 2006) might reflect the historical existence of more substantial forms of cheating.

Perhaps more important for maintaining group discipline, however, is the intricate complex of rites and rituals that have developed around the water temples. These rites and rituals are examples of group-level traits “…not expressed by any single individual in the group, but (which) emerge from the structured organization of different individuals” (Smaldino 2014: 244). Importantly, rituals, institutions, and the maintenance of water temples can entail significant time and effort and impose a heavy financial burden on households (Lansing 2006). As Lansing (2006: 126) notes, “Assembling the many varieties of offerings needed for the annual round of agricultural rites, to take just one example, imposes a relentless series of obligations on households…”

While some rites and rituals play a direct role in coordinating cropping (Lansing 1987, 2006), others have a more symbolic meaning, facilitating group solidarity and cooperation (Norenzayan and Shariff 2008; Watson-Jones and Legare 2016). The internalization of the ritual aspects of a system can reduce the need for costly enforcement and sanctioning (Atran and Norenzayan 2004; Sosis 2009; Sosis and Bressler 2003). These public displays address the challenges that the pursuit of self-interest can pose for a system that is so dependent on cooperation and coordination. Lansing (2006: 195) suggests that rituals also play a cognitive role by helping individuals transcend personal limitations and by instilling, “… a state of mind that is…different from that of *Homo economicus*.” As Lansing (2006: 136) writes, the purpose of the ritual displays is to help contain individual desires by “…mobilizing the powers of the collective to gain control…” (p. 137). These rituals address the very tension between individual self-interest and the good of the group that lies at the heart of understanding the evolution of cooperation.

### Organizational structure of the system

In this section, we examine how these two traits may have emerged and spread at multiple scales.

Three levels of social organization are important: (1) farmers/households, which are nested within (2) subaks, which are nested within (3) regional water networks (see Fig. 1). Whether farmers own, rent, or work farmland, they generally retain rights to what they produce (Lansing 2006) and bear the costs associated with synchronized cropping (i.e., sharing water, maintaining irrigation infrastructure, participating in and contributing resources to water temple rituals). Farmers are clustered into subaks, which can occupy 4 ha to over 800 ha (Scarborough et al. 1999) and include 50–400 farmers, frequently from different villages (Schoenfedler, 2001). Subaks coordinate cropping patterns and irrigation sequences and organize farmers to construct and maintain irrigation infrastructure (Lansing 1987). Subaks are an important first unit for regulating farmers’ behavior, but they are then clustered within regional water networks. One level of this network is an *Ulun Swi* temple, which serves several subaks whose canals are filled by a common weir or spring. Multiple *Ulun Swi* temples can be nested under *Masceti* temples, which unite a dozen or more subaks occupying a common stretch of river (Scarborough et al. 1999) (see Fig. 1). Finally, the main water temple, *Pura Ulun Danu Batur* sits at the edge of the volcanic lake that is the source of irrigation water for much of Bali (Scarborough et al. 1999). At this temple, priests arbitrate disputes and give approval and advice for the construction of new irrigation channels and tunnels. Subak members are summoned to this temple annually, where they make offerings to the water gods and receive guidance about when to start planting (Lansing 1987). While initial coordination occurs at the *Pura Ulun Danu Batur* temple, further coordination to synchronize planting times among member subaks occurs at *Ulun Swi* and *Masceti* temples at annual meetings of the elected subak leaders (Lansing 1987, 2006; Schoenfedler 2001). Thus, just as farmers cooperate and coordinate with other farmers within a subak, subaks also cooperate and coordinate with other subaks in the regional water network (Lansing 2006). The nested levels of decision making mirror the nested levels of physical irrigation works.

Lansing (2006: chapter 2) combines his own observations and ethnographic data with historic and archeological data (e.g., Scarborough et al. 1999) to convincingly argue that rice cultivation began in simple concave depressions, but increased in complexity to include large-scale coordination among the thousands of farmers in the region. In the first millennium a.d., Balinese farmers created channels and dug tunnels to irrigate terraced paddies on hillocks or “water mountains”. These engineered irrigation works required cooperation and coordination at increasingly larger scales as they connected villages downstream. As Lansing (2006: 42) writes:

“These systems of water control became more complex as new hillocks are added downstream. While a single water mountain is typically managed by farmers from one or two villages, several water mountains are often tethered to one or more irrigation systems, creating the need for water management at a larger scale.”

We suggest that the CMLS framework allows us to understand the emergence and growth of this system as the result of a shift in the dominant level of selection. When the dominant level of selection (e.g., group selection) is above the level of the social dilemma (e.g., individual farmers sharing water) selection can favor group-beneficial traits (Waring et al. 2015).

## The emergence of synchronized cropping, subaks, and associated rituals

While scholars (Lansing and Kremer 1993; Lansing et al. 2009) have modeled the emergence of clusters of synchronizing subaks (regional water networks), these models are based on the assumption that synchronized cropping and subaks already exist. But, there is little explanation for how synchronized cropping, subaks, or the institutions and rituals associated with subaks, emerged and spread. The CMLS framework suggests that selection for individually beneficial behaviors (e.g., withdraw as much water as I need) can operate at the same time, and in opposition to, selection for group-beneficial behaviors (e.g., share water and coordinate with other farmers in my subak). Based on the emergence of clear group structures and variation in the adoption of group-beneficial traits, cultural group selection could have become a stronger force and shaped the Balinese agro-ecological system. In the sections below, we use a hypothetical, but reasonable scenario to imagine how the dominant level of selection may have changed, how group structures may have emerged, and how cultural group selection mechanisms could have resulted in the spread of key cultural traits.

### Level of selection: farmers

Farming on Bali began between 4500 and 3000 years ago and farming communities and rice cultivation are estimated to have been present approximately 2600 years ago (Scarborough et al. 1999; Lansing 2006). As the area converted to paddy, cultivation increased, irrigation infrastructure expanded, and farmers faced the ecological constraints of water stress and growing pest populations (Lansing and de Vet 2012; Schoenfedler 2001). In the very early stages of Balinese agriculture, the dominant level of behavioral selection would likely have been the individual. Self-interested strategies would have been selected for because individuals who withdrew the most water would have produced higher yields and outcompeted those who did not.^1^

### Level of selection: subaks

**Fig. 2:**
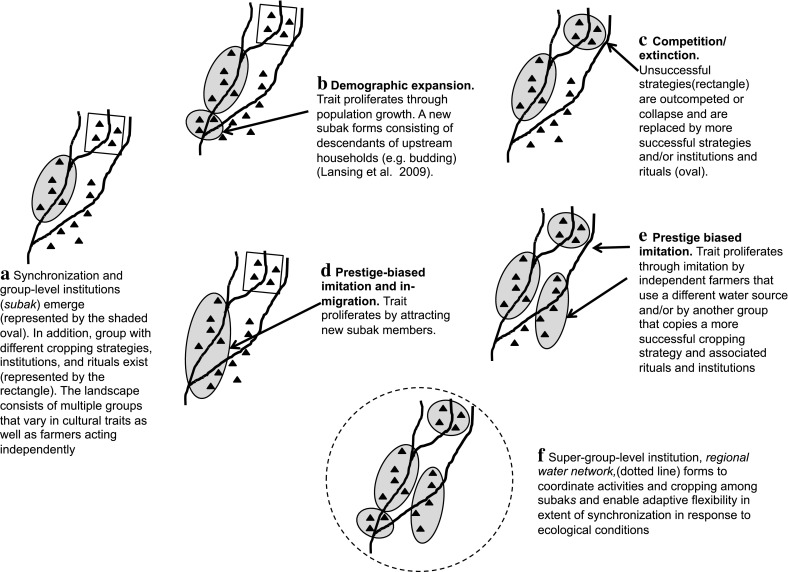
Group formation and cultural transmission mechanisms. Diagram **a** represents the emergence of groups with different cropping strategies, institutions, and rituals. Diagrams **b**–**f** represent mechanisms through which the traits of synchronized cropping and/or institutions and rituals could increase in the population. Diagrams **b**–**f** illustrate the proliferation of the trait and should be viewed in reference to the starting condition in diagram **a**

For group selection pressures to be stronger than individual selection pressures multiple groups must exist and there must be greater variation in the expression of a beneficial trait among groups than within them (Henrich 2004). To consider how selection pressures may have shifted to the group-level to favor group-beneficial traits, imagine a small group of farmers, who shared a water source. These farmers may have previously worked together to build and maintain small-scale irrigation infrastructure, which could have facilitated coordination efforts. Based on local knowledge about pest populations and expectations about the benefits of synchronization, this small group might have agreed to synchronize their cropping (see Fig. 2a). Alternatively, synchronization could have emerged “by accident” if several farmers independently copied the cropping pattern of a neighboring farmer who had produced the highest yield the previous year. (see Lansing et al. (2017) for a model of such a process) Given the autonomy over their agricultural practices (Lansing 2006; MacRae and Arthawiguna 2011), if synchronized cropping generated greater yields for farmers than they had experienced prior to synchronization, there would have been an incentive to continue synchronizing. The resulting group would have represented the earliest subak.

Other nearby farmers’ groups could have converged independently on synchronization or they could have developed other strategies for collectively managing pests and water. As long as individuals or groups were forming and experimenting this way, the landscape would have become populated with diverse strategies for water and pest management (Fig. 2a). This group-level variation is a necessary condition for cultural group selection.

As subaks developed and grew, the costs of coordination the incentives to free-ride would have grown as well, favoring the emergence and evolution of the rituals and institutions that we know today. Just as groups of farmers varied in their cropping patterns, there may also have been variation in the type and efficacy of institutions and rituals that emerged alongside them. If particular institutions or practices resulted in higher yields, they also could have been favored by cultural group selection. Indeed, there is evidence of variation in groups’ cropping strategies as well as in the institutions and rituals that emerged to facilitate collective action within the groups. For instance, Lansing (2006) writes that there “…was considerable variation in the ways that subaks governed themselves. Each subak was free to construct its own institutions and there was no external pressure to conform to a standard model” (p. 89). Furthermore “…the amounts of time and treasure devoted to these religious activities vary widely (among subaks)…” (p. 125). This variation also translated into different yields. For instance, Lansing (2006: 114) describes one dysfunctional subak whose “…harvests were dwindling…because the irrigation schedules were very disorganized”.

We do not want to imply that all subaks were converging on a single “ideal” set of institutions and rituals with identical components and organizational structures. Instead, several factors could maintain variation among subaks. For instance, complex institutions may be difficult to fully understand, leading to copying errors (Boyd and Richerson 2002; Nelson and Winter 1982) and spatial or temporal variation in local ecological and/or social conditions could lead to the development of slightly different institutional structures or rituals (Boyd and Richerson 2010; Smaldino 2014). Additionally, groups could continue to learn and experiment with new structures or features to improve overall outcomes (Boyd and Richerson 2010), Indeed, Lansing (2006: 89) refers to subaks as “ongoing experiments” and MacRae and Arthawiguna (2011) provide direct evidence of such experimentation. Just as continued variation within biological populations does not mean that selection is inactive, continued variation among groups does not mean group selection is inactive.

## Cultural group selection mechanisms

Before we outline the potential emergence and spread of higher level water networks, it is important to consider the cultural transmission mechanisms (Henrich 2004; Richerson et al. 2016) that might have increased the prevalence of the group-beneficial traits noted above. Although the strength of evidence varies, each of the processes outlined below could have facilitated the spread of synchronization and the associated rituals and institutions.

### Differential migration

Group-beneficial traits can increase in a population if individuals leave unsuccessful groups and migrate to groups that produce higher yields, adopting the norms and practices of the group when they arrive. Farmers might have been selective about where to establish their fields or could have moved residences, favoring locations with higher productivity as a result of synchronized cropping and effective institutions. Such movement would increase the prevalence of these traits in the population. While plausible, there is little evidence of such movement, in part because the mosaic of property rights (Lansing 2006) might have limited the ability of, or opportunity for, farmers to move to new land.

### Demographic expansion, competition, and group extinction

Reasonably assuming that greater yields support higher population growth, groups with synchronized cropping and effective institutions and rituals would have produced higher yields, supporting higher fertility. More rapid population growth could have lead to the establishment of new communities with the same cultural traits (Fig. 2b). Lansing et al. (2009) use genetic evidence to suggest that new subaks were created when demographic pressure forced descendants from growing villages to establish new settlements (and subaks) downstream. Farmers in these new subaks would likely have maintained synchronized cropping and the rituals and institutions from their “parent” community as a form of group-level vertical transmission.

Moreover, groups that obtain higher yields should also have more resources, allowing them to outcompete other groups. Direct competition, such as warfare, could lead to the “extinction” of the cultural traits of the losing group (Fig. 2c). While there is some evidence for competition among subaks (Lansing 2006, Chapter 4) it does not involve warfare. Instead direct conflict and warfare appears to have been much more common among royal families and lords (Hauser-Schäublin 2003; Lansing 2006) (see below). However, subaks can experience “mortality” even without direct external competition. For instance, Lansing (2006) notes the complete collapse of a subak as a result of poor leadership and lack of organization. The unique components of that Subak’s institutions and rituals would have been removed from the population upon its demise.

### Prestige-biased transmission

Prestige-biased transmission, or imitation of successful entities, could also have increased the prevalence of synchronized cropping in the population. Imitation can occur at the individual-level (Fig. 2d), with individuals copying another farmer’s successful cropping pattern or the subak level (Fig. 2e). In fact, Lansing et al. (2017) model the emergence of synchronization among farmers and Lansing and Kremer (1993) model a similar process among subaks to illustrate this mechanism. In the latter case, each subak checks the productivity of its four nearest neighboring subaks and adopts the cropping pattern of the neighboring subak with the highest yield. In as few as eight years, this decision rule leads to relatively stable clusters of subaks that have adopted the same cropping pattern (i.e. synchronization). By copying the most-successful neighboring group, subaks in the model increased their yields and promoted the spread of the most-effective cropping pattern for a particular location. Importantly, the clusters of synchronizing subaks that emerged in the model closely matched the patterns observed empirically.

## Level of selection: water temple networks

Using the model noted above, Lansing and Kremer (1993) suggest that imitation of successful subaks can produce adaptive regional water temple networks. However, even as regional water networks form, there remains a group-level social dilemma. While overall yields for a group of ten subaks might be highest when all subaks share water, the yield for any one subak might be higher if it cheated by withdrawing more water than agreed upon. Therefore, regional water networks also have mechanisms to punish rule breaking, including levying fines and shutting off water to an entire subak for a failure to fulfill its responsibilities (Lansing 2006). Moreover, the scale of coordination at the network level is significantly higher as there is now the need to ensure that guidance about crop varieties and irrigation timing is disseminated to hundreds of farmers across multiple subaks (Lansing and Kremer 1993).

Thus, institutional structures to facilitate cooperation and coordination among subaks could be selected (Fig. 2F). As multiple regional networks appear (structured around *Ulun Swi* and *Masceti* temples) the dominant level of selection (and the most important level of decision making) can again shift upward. Like subaks, emerging regional-level networks may have initially varied in the strategies they adopted to enable cooperation and coordination and disseminate information. The networks with institutions and rituals that best facilitated group-beneficial traits could have produced the higher yields. If so, these institutions and norms could have spread to other networks through prestige-biased imitation or the other cultural transmission mechanisms noted above (demographic expansion, direct competition, and migration). Importantly, these networks are adaptive in that the group of synchronizing subaks can change in response to ecological conditions. The scale of coordination required depends on rainfall, pest populations, and other constraints that are taken into account on an annual basis. Thus, in this system selection pressures could have favored institutions with mechanisms that would allow for quick response to changes in ecological conditions.

In the sections above, we have broadly outlined potential mechanisms for the spread of group-level traits. Together, archeological data, ethnographic information, and computer modeling support the explanation that the rice cultivation system in Bali grew in complexity through a process of cultural group selection operating at higher scales of social organization. Providing more precise details about how, and by whom, complex group-level traits are transmitted is more difficult. There are examples of groups that have imitated complex organizational structures, institutions, and rituals (even if incompletely and inaccurately) (Zefferman and Richerson 2014; Smaldino 2014; Norenzayan et al. 2016), but questions remain about how such complex institutions are transmitted between groups and this has been identified as an important area for future research (Smaldino 2014).

Through this scenario, we described how synchronized cropping could have first spread among subaks. Groups of subaks that integrated their agricultural system with existing religious beliefs and practices or groups of subaks in which cultural practices emerged in tandem with ecological practices/outcomes could have had higher levels of collective action. Then, as the number of subaks increased and the gains for higher levels of coordination emerged (as well as the threat of free-riding) another level of social organization was required. The bundle of traits (synchronization combined with religious institutions and practices) that generated the highest group benefits relative to individual costs could then have spread among regional networks through a number of cultural transmission mechanisms.

## Disruption in the level of selection: the nation and the individual

While we relied on a hypothetical scenario in the previous section, a recent event highlighted the impact of a change in the level of selection on the structure and function of the system. In the 1970s, the Indonesian government set a goal of becoming a rice-exporting nation (Lansing 2006). To accomplish this goal, Indonesia adopted green revolution reforms including the distribution of improved rice varieties and the heavy use of pesticides and agricultural innovations developed in different cultural and ecological contexts. Balinese farmers were told to ignore traditional cropping patterns and to plant as often as possible and those who did not adopt green revolution practices were called backwards and unpatriotic (Lansing 2006). As Indonesia began competing with other nations in global rice markets, lower-level group affiliations, practices, institutions, and rituals were weakened or abandoned.

The abandonment of the rituals, institutions, and norms that promoted collective action shifted the level of selection down from the subak level to the individual. After a few years of improved yields, farmers started suffering high levels of crop damage by pests and disease, which dramatically reduced both individual and overall rice productivity (Lansing 2006). Thus, centralized decision makers promoted technology and practices that ignored important group structures, institutions and rituals and were maladaptive in Bali’s ecological and cultural context. In the intervening years, the system has largely reverted to traditional practices.

## CMLS and the top-down perspective on water management

The argument that the system for water management in Bali emerged as a process of self-organization is well supported with a range of compelling evidence. However, other scholars have suggested that the system was historically controlled and shaped from the top-down by royal elites (Hauser-Schäublin 2003; Schulte Nordholt 2011). While a full review of the evidence for both perspectives is beyond the scope of this paper, we argue that the CMLS framework can also be used to understand how such a system could have emerged through top-down forces and selection pressures.

**Fig. 3:**
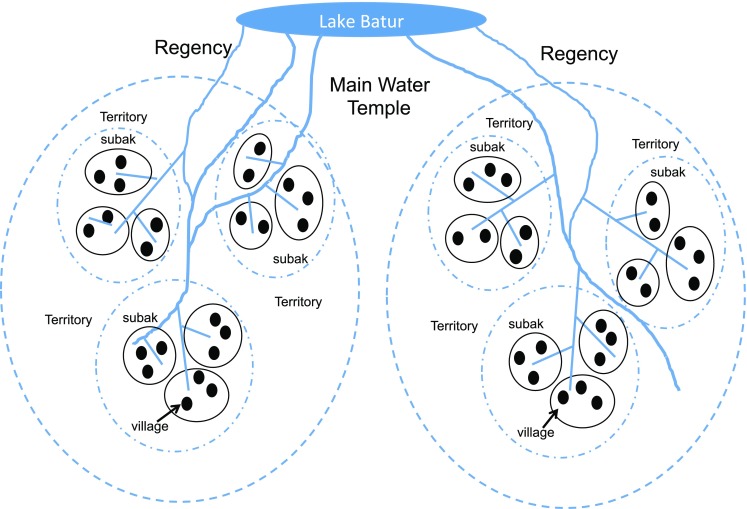
Group structure from the top-down perspective. Simplified diagram of the organizational structure for coordinating wet-rice cultivation in Bali from the top-down perspective. Subaks can include multiple villages, exist within territories and regencies, and are controlled by lords and kings

Some scholars have argued that kings, lords, and members of the nobility were directly involved in water management and the construction and maintenance of dams (Hauser-Schäublin 2003; Schulte Nordholt 2011). Politics and religion were closely connected and water temples were described as political tools that could be harnessed by royal families (Hauser-Schäublin 2003, 2005). Kings organized rituals, made decisions about water use and distribution, and adjudicated disputes among subaks. In addition, regional water networks were linked to powerful clans and nobility nested within regencies who competed for the allegiance of villages (Hauser-Schäublin 2003). From this top-down perspective, the most prominent group structures and, subsequently, the key levels of selection, would have been: (1) nine regencies, each controlled by a king, (2) territories embedded within these regencies and controlled by lords and noble families, (3) subaks within territories, (4) villages within subaks, and (5) individuals within villages (see Fig. 3, individuals not pictured).

A king’s authority was derived from his ability to command subjects to join an annual pilgrimage to honor important deities (Hauser-Schäublin 2003, 2005). These pilgrimages made visible the boundaries of the regencies and territories and provided an indicator of the size of the population that was loyal to a lord or king (Hauser-Schäublin 2003). The pilgrimages also strengthened social bonds and a sense of community (Hauser-Schäublin 2005), which can be important for collective action. Allegiance to a king was partly based on the degree to which he was perceived to channel the power of the gods to ensure the fertility of the populace and their crops (Hauser-Schäublin 2003, 2005). A king’s power thus reflected the productivity of the fields under his control and, by extension, wise water management. High yields would strengthen the allegiance of subjects to their ruler and provide greater tax revenue and contributions towards important rites and rituals. Lords and nobles would have been similarly motivated to manage water and cropping patterns to maximize overall yields because they competed with each other for lands, the allegiance of peasant farmers, and the favor of the king.

Unfortunately, there is little discussion in this literature about how top-down control of irrigation emerged. It is unlikely that kings actively developed this complex system from scratch given the layered, system-wide understanding that this would have required (Pedersen 2005). Instead, we can imagine another hypothetical scenario that begins with a small group of farmers experimenting with synchronized cropping and that grows to the point of being harnessed by the governing groups that are incentivized to ensure the highest yields among their subjects.

The same cultural group-selection mechanisms outlined above would apply to each level in the hierarchy here. Subaks, territories and regencies where synchronization was common could have grown more rapidly (demographic expansion), attracted more households (migration), been imitated by other groups (prestige-biased transmission), or outcompeted other groups (direct competition/warfare). In addition, because lords and kings had an incentive to identify practices that increased yields, they could have actively promoted the spread of synchronized planting across subaks, territories, and/or the regency under their control (akin Durham’s (1991) concept of ‘imposition’).

In the top-down narrative, direct inter-group competition among royal families, lords, and powerful clans would likely have been a more important driving force for cultural evolution. Kings and lords would have competed for the allegiance of villages and the productive land they worked. Rulers who managed irrigation water and who harnessed the power of religion and ritual would have been more effective at generating cooperation within and among subaks and would thus have earned greater tax revenue from the higher yields of subaks they controlled. The tax revenue could have been used to field stronger armies or to win status races. A growing population would have increased the need for agricultural land, which could have contributed to inter-group competition and warfare. For instance, Hauser-Schäublin (2003) refers to several early studies on Bali that indicate frequent conflict and warfare. In addition, Lansing (2006: 57) writes, “The main area for conflict was the southern rice bowl of Bali, where by the eighteenth century little kingdoms appeared and disappeared on the time scale of decades.” More productive regencies would have earned higher tax revenues and had more resources and a larger population upon which to draw in times of war (Hauser-Schäublin 2003, 2005), allowing them to outcompete other lords.

In short, the CMLS framework can still inform the argument that subaks were not completely autonomous and independent of top-down control by kings. Using the CMLS framework to differentiate between the two arguments requires identifying (1) which group structures are most relevant by determining the groups (subaks or territories) farmers most strongly identify with, and (2) which transmission mechanisms are most relevant. While there is more compelling evidence in support of the bottom-up perspective on the emergence of subaks (see commentary in Hauser-Schäublin 2003; Lansing 2005; Lansing and de Vet 2012; Pedersen 2005), it is not necessary to adopt an either/or perspective (Pedersen 2005) or presume that bottom-up and top-down forces acted independently. Presenting both arguments allows us to demonstrate how the CMLS framework informs both explanations by treating top-down and bottom-up pressures not as categories, but as forces that influence the likelihood that key behaviors will be adopted by, for instance, altering the costs and benefits of those behaviors.

## Discussion and conclusion

We have applied the CMLS framework to a well-known, complex social–ecological system to show how the framework can be used to understand the emergence and persistence of a sustainable social–ecological system. To this end we combined evidence from the literature with hypothetical scenarios to discuss: (1) the emergence of group structure, (2) variation in cropping strategies, institutions, and rituals adopted by groups, (3) how variation in yields as a result of different strategies, institutions, and rituals could have created the conditions for group selection pressures to outweigh individual selection pressures, and (4) the cultural transmission mechanisms that can explain the spread of group-beneficial traits. We also note that Lansing’s description of the function of water temples and rituals mirrors the persistent tension between individual self-interest and group-level benefits.

In applying this framework, we also sought to demonstrate how the CMLS framework complements previous explanations for the emergence of the Balinese system. Lansing and colleagues have provided convincing evidence that groups of farmers, water temples, and associated decision-making institutions in Bali constitute a complex adaptive system (CAS) that emerged through a process of self-organization (Lansing 2006). To complement the CAS perspective, the CMLS framework provides insights into specific cultural transmission mechanisms that can explain the role selection played in the development of increasingly complex structures. Several scholars have noted that, rather than being in conflict, CAS and evolutionary theory are complementary, in part because attention to selection pressures is needed to understand the development of increasingly complex structures (Hodgson and Knudsen 2010; Kauffman 1993; Levin 1998). For instance, Hodgson and Knudsen (2010: 55) write, “self-organization explains neither the characteristics of the elements that interact to create the emergent order, nor how the emergent order adapts and survives in the broader environment”. In the Balinese case, it is important to remember that there are still selection pressures acting on individual farmers within subaks and shaping group structures over time.

The CMLS framework is agnostic about sustainability. It does not suggest that, given enough time, cultural evolution will necessarily promote long-lasting social–ecological systems. Forces from multiple levels of organization may not push in the same direction in all systems or at all times. The green revolution era was evidence of this in Bali, and external forces and/or internal policies have shifted the dominant level of selection in deleterious ways in other systems (see examples in Waring et al. 2015). Instead the value of CMLS is that it provides a framework for examining the impact that particular policies or interventions, understood as driving forces of change, can have on a system and whether they are likely to result in more cooperation or more self-interested behavior.

Importantly, our analysis is necessarily retrospective. We have relied on published historical, ethnographic and archeological data and logic to surmise how this system might have emerged and spread. However, the CMLS framework can also be prospective; it can be used to analyze current trends and develop hypotheses, as other papers in this special issue illustrate (Kline et al., Hillis et al.). If we could study the system as it was first emerging and evolving, we would focus on several pieces of evidence about the relative costs and benefits of different agricultural practices, the salience of different group structures, and other important aspects of the system. Using the example of Bali's agro-ecological systems, we conclude by highlighting the data that would be required to fully test hypotheses derived from the CMLS framework about the evolution of social–ecological systems:

- Measures of the perceived individual costs and individual and group-level benefits of synchronized cropping relative to other cropping patterns and water management strategies (i.e., staggered planting). This should be done for different farmers in different communities (upstream versus downstream) and in years with different ecological conditions (high vs. low rainfall and high vs. low pest threat). Information on perceived net benefits at different scales could then be used to determine the likelihood that group selection pressures will be stronger than individual ones and whether a group-beneficial trait can spread as a result.
- Information on the salience of group identities and the degree to which the subak is the most important social group for farmers (particularly in comparison to the territory or regency in which a farmer lives). Because individuals can belong to multiple social groups simultaneously (i.e., a kin group, a village, a subak, and a regional water network), data that sheds light on the salience of these groups in particular contexts can illuminate whether group selection pressures are likely to act on that group. If farmers note that being part of a subak is more important socially than identifying with where they live, then group selection pressures would be more likely to operate on the subak than on the village or territory.
- Measures of variation in synchronized cropping and correlations between synchronization and agricultural productivity among subaks as well as measures of the degree of variation within groups versus between groups. Group selection pressures are more likely to operate on traits if there is variation among groups in the expression of those traits and that variation is highly correlated with rice productivity.
- Information on the mechanisms for monitoring and enforcing water use and water use agreements, the frequency of water theft, and the costs of punishment. This data could indicate the degree of conflict and competition among groups as well as the individual and group-level costs of maintaining cooperation.
- Measures of geographic variation in water availability and other ecological and topographic features that may relate to expression of the cultural trait, salient group identities, and institutional features (e.g., irrigation infrastructure, water temple rituals and ceremonies) related to water management.
- Tracking different cultural group selection mechanisms by examining social interactions across groups that could facilitate social learning and imitation, levels of competition among groups, migration patterns, and variation in population growth rates.

Historical reinterpretations of the type presented here provide an important first step in clarifying the important features and causal mechanisms that should receive close attention in the future. However, empirical work will be needed for more direct examination of the value and validity of the CMLS framework for understanding how sustainable social–ecological systems emerge, persist, and spread.

## Footnotes

1 There can be a high degree of genetic relatedness within and between subaks (Lansing et al. 2009), supported by endogamous marriage (Lansing 2006). Therefore, kin-selection may partially explain levels of cooperation. However, villages vary in their degree of genetic relatedness (Lansing et al. 2009), and subaks have been described fragile institutions prone to breakdown despite interactions among kin (Lansing 2006). Moreover, the extent of cooperation and coordination required across the system likely goes beyond that which can be explained by kin selection alone (Henrich 2004).
